# Genome Selection for Fleece Traits in Inner Mongolia Cashmere Goats Based on GWAS Prior Marker Information

**DOI:** 10.3390/ani15213184

**Published:** 2025-10-31

**Authors:** Huanfeng Yao, Na Wang, Yu Li, Gang He, Jin Ning, Shuai Kang, Yongbin Liu, Jinquan Li, Qi Lv, Ruijun Wang, Yanjun Zhang, Rui Su, Zhiying Wang

**Affiliations:** 1 College of Animal Science, Inner Mongolia Agricultural University, Hohhot 010018, China; yaohuanfeng@emails.imau.edu.cn (H.Y.); ybliu@imu.edu.cn (Y.L.); lvqi1202@imau.cn (Q.L.); imauwrj@126.com (R.W.); imauzyj@163.com (Y.Z.); suruiyu@126.com (R.S.); 2 Inner Mongolia Yiwei White Cashmere Goat Co., Ltd., Ordos 017000, China; wangn@163.com (N.W.); liyu@163.com (Y.L.); 13015206915@163.com (G.H.); xihaijiao456@163.com (J.N.); 13284371747@163.com (S.K.); 3 Inner Mongolia Key Laboratory of Sheep & Goat Genetics Breeding and Reproduction, Hohhot 010018, China; lijinquan@163.com; 4 Key Laboratory of Mutton Sheep & Goat Genetics and Breeding, Ministry of Agriculture and Rural Affairs, Hohhot 010018, China

**Keywords:** prior information, genomic selection, fleece traits, Inner Mongolia cashmere goats

## Abstract

**Simple Summary:**

Integrating prior marker information into genomic selection models can significantly enhance the genomic prediction accuracy of fleece traits in Inner Mongolia cashmere goats. In this study, a genome-wide association study was conducted on these fleece traits, and the resulting prior SNPs were weighted and then integrated into a new genomic relationship matrix to estimate genomic prediction accuracy. The results indicate that, compared with the conventional genomic best linear unbiased prediction model, the method that integrates prior markers for genomic evaluation can significantly improve the genomic prediction accuracy of fleece traits. This improvement provides valuable support for the more precise estimation of genomic breeding value in Inner Mongolia cashmere goat breeding programs.

**Abstract:**

The Inner Mongolia Cashmere goat (IMCG) industry is a major contributor to global cashmere production, with fleece traits serving as key economic indicators that directly impact both income and the long-term sustainability of the industry. When genome-wide SNPs are used to estimate kinship matrices, the traditional animal model implicitly assumes that all SNPs have the same effect-size distribution. However, in practice, there are differences in the genetic mechanisms and complexity of different traits. We conducted a genome-wide association study (GWAS) on 2299 IMCGs genotyped with 67,021 SNPs, which were obtained after imputation. The traits measured included cashmere yield (CY), wool length (WL), cashmere length (CL), and cashmere diameter (CD), with a total of 33,564 records collected. The top 5% to 20% of the significant SNPs from the GWAS were used as biological prior information. We then assigned proportional weights based on their contribution to the overall genetic variance and further integrated them with the remaining loci to construct a kinship relationship matrix for estimating genetic parameters and genomic breeding value. By incorporating prior marker information from the GWAS, it was found that the heritability estimates for CY, WL, CL, and CD were 0.26, 0.37, 0.09, and 0.35, respectively. For CY and CL, integrating the top 5% of prior SNP markers yielded the highest genomic prediction accuracies of 0.742 and 0.673, representing improvements of 16.67% and 19.75% over models that did not utilize prior information. In contrast, for WL and CD, the highest accuracies of 0.851 and 0.780 were achieved by integrating the top 10% of prior SNP markers, reflecting improvements of 9.81% and 10.14%, respectively. Compared with the conventional GBLUP method, this method of integrating GWAS-derived prior markers for genomic genetic evaluation can significantly improve the accuracy of genomic prediction for fleece traits in IMCGs. This approach facilitates accurate selection for fleece traits in IMCGs, enabling accelerated genetic progress through long-term breeding programs.

## 1. Introduction

The Inner Mongolia cashmere goat (IMCG), a genetically improved dual-purpose breed, is distinguished by its superior fiber quality, high cashmere yield, and gourmet-quality meat attributes. As a key genetic resource in China, this breed significantly contributes to the sustainability and stability of the global cashmere industry [1]. Fleece traits serve as primary economic indicators in cashmere goat production, critically influencing farm profitability and the sustainable development of the cashmere industry. The annual amount of cashmere produced globally is approximately 20,000 tons, with China being the main producer, accounting for 75% of the annual output [2]. Given the rising global demand for superior cashmere, genomic selection (GS) and other molecular breeding technologies have emerged as pivotal research priorities in genetic improvement programs.

Traditional breeding methods, relying predominantly on phenotypic and pedigree-based selection, have achieved certain levels of genetic improvement. However, their efficacy remains constrained by low selection accuracy and modest genetic gains, particularly for low-heritability traits. Rapid progress in molecular genetics and genomics has facilitated the implementation of advanced breeding technologies, including genome-wide association studies (GWASs) and GS, providing novel strategies for the genetic enhancement of livestock. GWASs serve as a powerful tool for identifying candidate genomic loci linked to economic traits through systematic evaluation of associations between SNPs and phenotypic variation. The derived *p*-values and effect size estimates from GWASs not only yield critical biological insights but also function as informative priors in animal models for GS, substantially improving their predictive performance. Empirical evidence demonstrates that integrating such prior knowledge markedly enhances the accuracy of genomic predictions [3,4,5].

The animal model within the genomic best linear-unbiased prediction (GBLUP) method operates under the assumption that all SNPs contribute equally to genetic variance, with normally distributed effects. This model offers notable flexibility, as it can incorporate multiple genomic relationship matrices (G-matrices) and allows for differential weighting of these matrices [6]. To overcome the constraints imposed by uniform SNP effect assumptions, researchers have developed enhanced animal models in genomic selection that integrate prior biological information. A notable innovation in this area is the Genomic Feature Best Linear Unbiased Prediction (GFBLUP) method [7]. This approach maps marker information to gene ontology and integrates several genomic feature classes that can be formed based on different sources of prior information. It provides a flexible way to incorporate various genomic features and external information, thereby offering an advanced analytical framework for elucidating the genetic architecture of complex traits. BayesRC [8] incorporates prior biological information into the analysis by categorizing the contribution of genetic variants to trait heritability, such as variant annotation, candidate gene lists, and known causal variants. This approach is considered to improve the accuracy of genomic prediction. Zhang et al. [9] proposed the BLUP|GA method, which calculates the contribution of individual loci to genetic variance based on GWAS results. This information is then used to assign weights to a trait-specific genomic relationship matrix, replacing the traditional G matrix. This approach effectively utilizes information on the genetic architecture of the trait while also reducing computational burden, thereby improving prediction accuracy and outperforming both the GBLUP and BayesB models [10]. Similarly, the MultiBLUP method introduces multiple random effects to assign different effect size variances to different categories of SNPs or to integrate various omics data, thereby greatly enhancing the predictive power for complex traits [11]. Lopes et al. [12] improved the prediction of teat number in pigs by integrating GWAS-significant SNPs as fixed effects in marker-assisted BLUP (MA-BLUP) and marker-assisted genomic BLUP (MA-GBLUP) models. Notably, MA-GBLUP demonstrated superior performance over conventional GBLUP, particularly in scenarios involving small training populations or distant validation populations. Substantial evidence now supports the conclusion that integrating prior biological knowledge into GS models can markedly improve the accuracy of genomic predictions, particularly for complex traits [13,14,15,16]. These findings collectively underscore that incorporating prior biological information is an effective strategy for optimizing genomic prediction models. This integration not only enhances prediction accuracy but also provides a solid theoretical foundation and practical framework for modern animal breeding.

We aimed to evaluate the accuracy of genomic prediction for fleece traits in IMCGs by constructing a weighted G-matrix using GWAS-derived prior marker information. We anticipate that this approach will enhance both the accuracy and application efficiency of genomic selection in cashmere goat breeding programs. The findings are expected to provide a theoretical foundation and technical support for genetic improvement in the cashmere goat industry.

## 2. Materials and Methods

### 2.1. Phenotypic Data Sources

In this study, 2299 IMCGs (Arbas type) were sampled from the breeding farm of Inner Mongolia Yiwei White Cashmere Goat Co., Ltd., located in Ordos, Inner Mongolia, China. A total of 33,564 phenotypic records were collected from IMCGs aged of 1 to 8 years, encompassing four key fleece traits. Among them, for the cashmere yield (CY), Wool length (WL), cashmere length (CL), the average number of records per individual is approximately 4.26 times, and more than 74% of individuals have records of three or more times. For the cashmere diameter (CD), the average number of records per individual is about 2.42 times, and more than 49% of individuals have records of three or more times. The data covers key stages of the individual’s growth cycle. Preliminary statistical analyses of the phenotypic data were performed using Microsoft Excel and R [17] software. Following standard quality control procedures, outliers exceeding the mean ± 3 standard deviations were identified and excluded, while the remaining high-quality data were retained for next analyses.

Since GWASs have limitations in pinpointing mutation sites controlled by polygenic effects with small individual contributions, the lme4 [18] package in R (Version: 4.3.1) was employed to adjust for the effects of measurement year, herd, sex, and individual age on phenotypic values of fleece trait. The Best Linear Unbiased Estimates (BLUE) for each fleece trait were obtained and used as adjusted phenotypic values for subsequent GWAS analysis [19]. All traits were found to follow a normal distribution after phenotype adjustment.

### 2.2. Genotypic Data and Quality Control

The genotypic data used in this study consisted of imputed 1X sequencing data from 2299 IMCGs. The 1X sequencing data were randomly selected from the 10X high-coverage sequencing data as part of the research group’s previous work [20]. Quality control was implemented in PLINK v1.90 [21] with the following thresholds: minimum allele frequency (MAF) < 0.01, Hardy-Weinberg equilibrium *p*-value < 1 × $10^{-6}$ , and an SNP call rate < 0.90 for individuals. The retained loci were used for subsequent analysis. After quality control of the 1X resequencing data for IMCGs, 67,021 SNPs were retained for GWAS and GS. It was showed that no significant population stratification was obtained among the 2299 IMCGs through PCA analysis.

### 2.3. Selection of Prior Information Derived from GWAS

GWAS were performed using GAPIT 3 [22] package in R. For each fleece trait, we conducted GWAS employing Linkage-disequilibrium Iteratively Nested Keyway (BLINK) model [23]. The BLINK model, by integrating Bayesian information with linkage disequilibrium information, can effectively correct for population stratification effects. It significantly reduces computation time while controlling for false positives. The formula of the BLINK model is presented below:

(1) $$y_{i}=S_{i1}^{*}b_{1}+S_{i2}^{*}b_{2}+\cdots +S_{ik}^{*}b_{k}+S_{ij}d_{j}+e_{i}$$

(2) $$y_{i}=S_{i1}^{*}b_{1}+S_{i2}^{*}b_{2}+\cdots +S_{ik}^{*}b_{k}+e_{i}$$

(3) $$BIC=-2LL+2KLn\left(n\right)$$

The BLINK method involves two fixed effect models. In the Formula (1), $y_{i}$ is the phenotype of the ith individual; $b_{1}$ , $b_{2}$ , …, $b_{k}$ , are the vector of the kth pseudo QTNs effect; $S_{i1}^{*}$ , $S_{i2}^{*}$ , …, $S_{ik}^{*}$ is the structure matrix of individual; $S_{ij}$ is the genotype of the *j*th SNP, $d_{j}$ is the effect of the j th SNP; $e_{i}$ is the residual effect. In the Formula (2), based on the BIC [24], the best t pseudo QTNs are selected from the k pseudo QTNs, and the process continues until no new pseudo QTNs are identified or the maximum number of iterations is reached. In the Formula (3), LL represents the log-likelihood value; K is the number of pseudo QTNs; Ln denotes the natural logarithm; and n is the number of individuals.

Each SNP is treated as a fixed factor in the regression analysis, followed by a significance test to calculate the *p*-value for each SNP. Subsequently, the top 5%, 10%, 15%, and 20% of SNPs with the smallest *p*-values are selected. These SNPs are then weighted according to their contributions to additive genetic variance and utilized as prior marker information for GS.

### 2.4. Genomic Selection by Integrating GWAS Prior Marker Information

In this study, the mixed linear animal model was constructed to perform the estimates of variance components and genetic parameters for fleece traits in IMCGs by ASRgenomics package in ASReml [25]. The GBLUP model utilized in this study is outlined as follows:

y=Xb+Zu+Wp+e

where y is the observed value vector of each trait, b is the vector of fixed effect, including herd, measurement year, individual age, and sex. u is the vector of individual additive genetic effect, u ~ N (0, Gt $\sigma_{g}^{2}$ ). *p* is the vector of permanent environmental effects, p ~ N (0, I $\sigma_{p}^{2}$ ). X , Z and W are the structural matrices of fixed effect, individual additive genetic effect and permanent environmental effects, respectively. e is the vector of residual effect, e ~ N (0, I $\sigma_{e}^{2}$ ).

The kinship matrix utilized in GBLUP was proposed by VanRaden [26]. Generally, the G-matrix is a genomic relationship matrix commonly used in genomic prediction to represent genomic similarity between individuals. The G-matrix is defined as follows:

$$G=\frac{ZZ^{T}}{2\sum_{i=1}^{m}p_{i}\left(1-p_{i}\right)}$$

Generally, the GBLUP assumed that the contributions of each marker to genetic variance is equal, but this hypothesis was considered to violate in practice due to heterogeneous of the effects among markers. Therefore, we partitioned all SNP markers into two subsets: prior-information markers ( $G_{1}$ ) and remaining markers ( $G_{2}$ ). The genetic variance of the trait in $G_{1}$ and $G_{2}$ is then calculated, and a new matrix $G_{t}$ is formed by weighting the proportion of explained genetic variance. The equation of $G_{t}$ is as follows:

$$\begin{array}{c} G_{t}=\omega G_{1}+\left(1-\omega \right)G_{2} \end{array}$$

The $G_{1}$ was genomic relationship matrix constructed by using the top 5%, 10%, 15%, and 20% of SNP loci selected from the GWAS results. The $G_{2}$ was genomic relationship matrix constructed by using the t the remaining markers. $\omega =\frac{\sigma_{G_{1}}^{2}}{\sigma_{G_{1}}^{2}+\sigma_{G_{2}}^{2}}$ , with $\sigma_{G_{1}}^{2}$ and $\sigma_{G_{2}}^{2}$ representing the genetic variances of $G_{1}$ and $G_{2}$ matrices, respectively.

### 2.5. Evaluation of Genomic Prediction Accuracy

In this study, the five-fold cross-validation was employed to evaluate the accuracy of genomic prediction under an animal model incorporating GWAS-derived prior marker information. Specifically, the population in this study was randomly divided into five subgroups. One subgroup was designated as the validation population (individuals with genotypes only), while the remaining four subgroups, comprising individuals with both genotypic and phenotypic data, were as the reference population. This process was repeated five times in a circular manner, ensuring each subgroup served as the validation set once. Prediction accuracy was assessed by computing the mean Pearson correlation coefficient between genomic estimated breeding values (GEBV) and observed phenotypic values across all five repetitions [27]. A correlation coefficient closer to 1 indicates higher prediction accuracy. The formula was as follows:

$$r=\frac{cov\left(a,p\right)}{\sqrt{var\left(a\right)var\left(p\right)}}$$

where a is the genomic estimated breeding values, and p observed phenotypic values.

In addition, multiple comparisons [28] was performed to analyze the effect of incorporating different proportions of GWAS-derived prior marker information on the genomic prediction accuracy for fleece traits in IMCGs.

## 3. Results

### 3.1. Selection of Prior Information Datasets from GWAS

**Figure 1:**
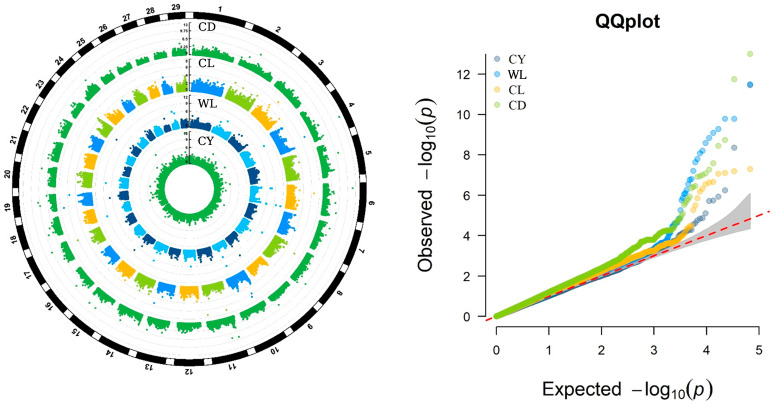
Manhattan Plots and QQ-plots showing GWAS results for four fleece traits in IMCGs. CY = cashmere yield; WL = Wool length; CL = cashmere length; and CD = cashmere diameter.

In this study, genome-wide association analyses for fleece traits were performed using the BLINK model. The results, including the Manhattan and Q-Q plots were shown in Figure 1, demonstrate a well-fitted Q-Q plot, indicating the model’s validity and the reliability of the findings.

**Figure 2:**
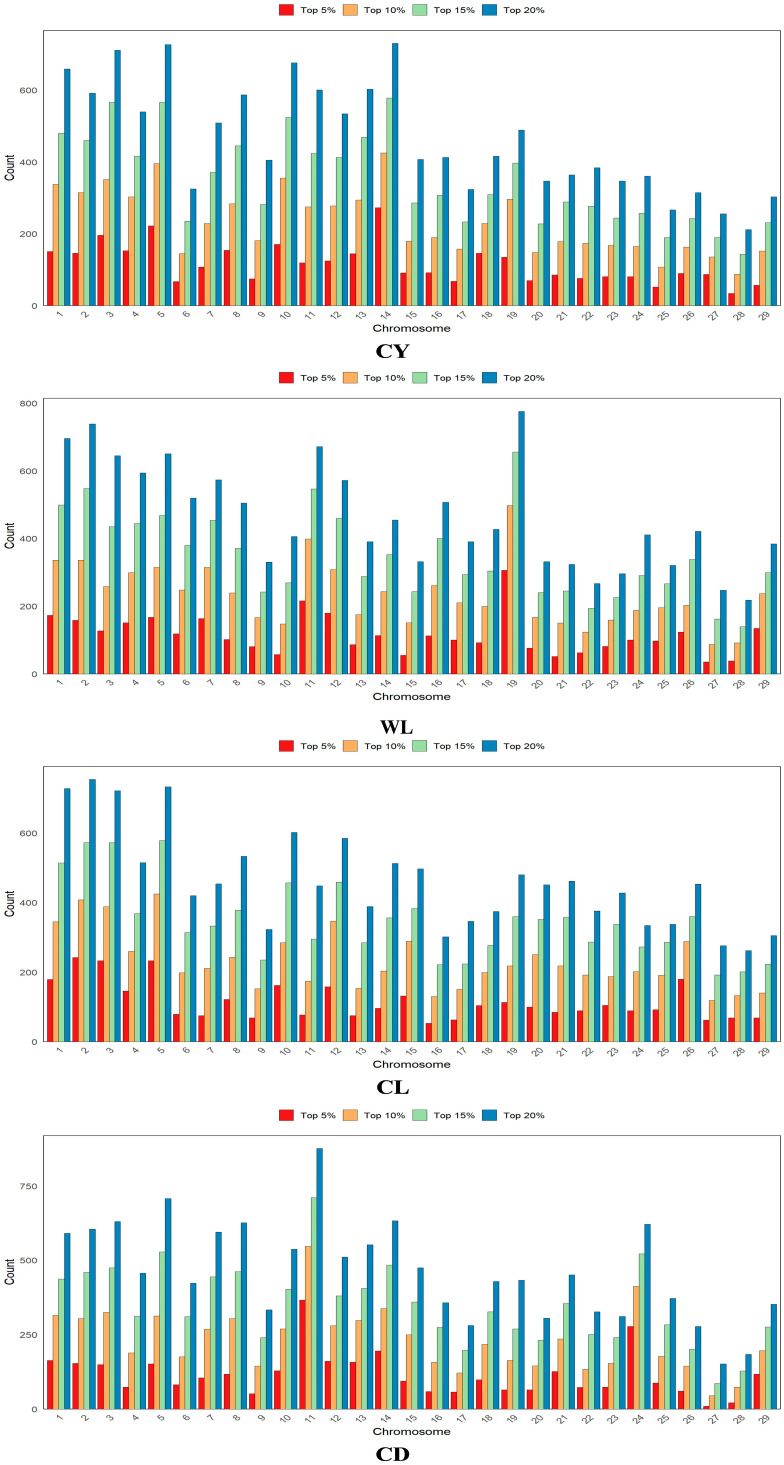
Distribution of fleece trait-related prior information on chromosomes. CY = cashmere yield; WL = Wool length; CL = cashmere length; and CD = cashmere diameter.

Based on the GWAS results, SNPs were ranked according to their *p*-values, and the top 5%, 10%, 15%, and 20% were selected as prior marker information for GS analysis. Specifically, the top 5% included 3351 SNPs, the top 10% encompassed 6702 SNPs, while the top 15% and 20% consisted of 10,053 and 13,404 SNPs, respectively. The chromosomal distribution of fleece trait-related prior information is illustrated in Figure 2, highlighting the variation in SNPs associated with the genetic control of each trait.

### 3.2. Estimates of Genetic Parameters of Fleece Traits in IMCGs

According to the estimation results of genetic parameters for CY in IMCGs (Table 1), as the proportion of prior SNPs selected from GWAS increased from the top 5% to the top 20%, the genetic variance estimated using the G_1_ relationship matrix increased from 8949.80 to 9934.10, and the corresponding heritability rose from 0.23 to 0.26. Meanwhile, the weight assigned to G_1_ increased from 0.60 to 0.75. In contrast, the genetic variance of the G_2_ matrix, constructed from the remaining SNPs, decreased from 5945.97 to 3254.63, with heritability dropping from 0.15 to 0.08. The weight of G_2_ also declined from 0.40 to 0.25. The permanent environmental variance for G_1_ showed little change, while that of G_2_ continued to increase. By integrating the top 5% to top 20% prior SNPs to construct the G_t_, the total genetic variance increased from 9757.60 to 10,040.40, the permanent environmental variance does not show a consistent pattern. The estimated heritability was 0.26, and the repeatability across all test sets remained stable at approximately 0.33, indicating consistent model performance.

According to the estimation results of genetic parameters for WL in IMCGs (Table 2), as the proportion of prior SNPs selected from GWAS increased from the top 5% to the top 20%, the genetic variance estimated using the G_1_ relationship matrix remained relatively stable (ranging from 7.33 to 7.55), with heritability consistently around 0.37 and weight increasing from 0.60 to 0.72. In contrast, the genetic variance of the G_2_ matrix decreased from 4.80 to 2.87, with heritability declined from 0.24 to 0.15 and weight decreasing from 0.40 to 0.28. Similarly, the permanent environmental variance for G_1_ showed little variation, while that for G_2_ increased steadily. The matrix G_t_, constructed by integrating the top 5% to top 20% prior SNPs, was used to estimate the total genetic variance increased from 7.31 to 7.41 and then slightly declined, while the permanent environmental variance rose from 1.92 to 2.20. The heritability estimated from G_t_ remained approximately 0.38, and the repeatability across all test sets was stable at around 0.48.

According to the estimated genetic parameters for CL in IMCGs (Table 3), the genetic variance derived from the G_1_ relationship matrix remained approximately 0.10, with a heritability of around 0.09. while the proportion of variance explained by G_1_ increased from 0.61 to 0.80. For the G_2_ matrix, the genetic variance decreased from 0.06 to 0.02, and the corresponding heritability declined from 0.06 to 0.02. The proportion of variance attributed to G_2_ also decreased from 0.39 to 0.20. The permanent environmental variance of G_1_ remained close to zero, while that of G_2_ showed an increasing trend. Using the combined G_t_, which integrates prior marker information from the top 5% to top 20% SNPs, the genetic variance was estimated at 0.10, with a permanent environmental variance of approximately zero and heritability around 0.09. Repeatability across all test sets remained stable at approximately 0.08.

According to the genetic parameter estimates for CD in IMCGs (Table 4), the genetic variance derived from the G_1_ relationship matrix remained approximately 0.18, with a corresponding heritability of around 0.35. The weight assigned to G_1_ increased from 0.61 to 0.74 across these thresholds. In contrast, the G_2_ matrix exhibited a decrease in genetic variance from 0.12 to 0.06 and a reduction in heritability from 0.23 to 0.12, with its assigned weight decreasing from 0.39 to 0.26. The permanent environmental variance for G_1_ remained close to zero, while that of G_2_ showed an increasing trend. When integrating the top 5% to 20% prior SNPs into a combined G_t_ matrix, the estimated genetic variance was approximately 0.17, with a near-zero permanent environmental variance and a heritability estimate of 0.35. The repeatability across all validation sets ranged from 0.34 to 0.37, indicating stable model performance.

**Figure 3:**
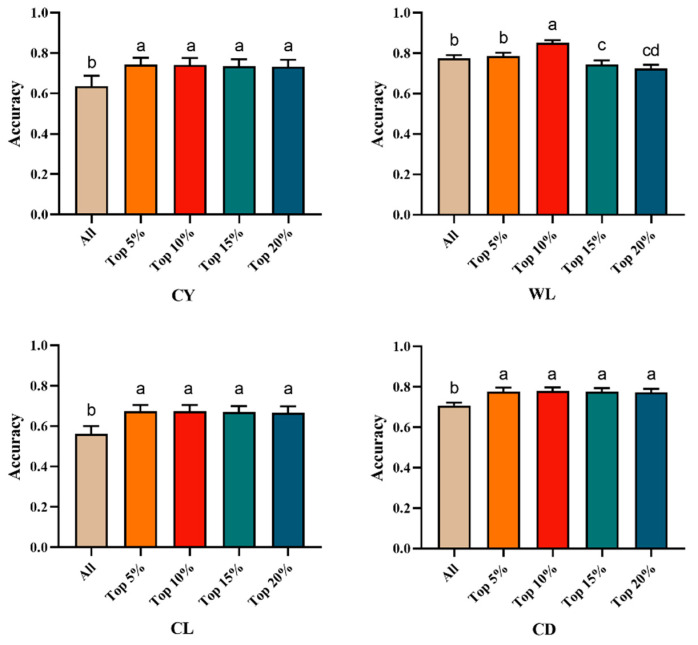
Multiple comparison plots of genome prediction accuracy for fleece traits with integrated Top prior information. Different letters indicate a significant difference at *p* < 0.05, and the same letters indicate no significant difference at *p* > 0.05. CY = cashmere yield; WL = wool length; CL = cashmere length; and CD = cashmere diameter.

By incorporating the top 5% to 20% of GWAS-identified SNPs as prior marker information into the genomic relationship matrix (Table 5 and Figure 3), the genomic prediction accuracy for CY ranged from 0.732 to 0.742, representing an improvement of 15.09% to 16.67% over the traditional GBLUP method. For WL, the genomic prediction accuracy with GWAS-informed prior markers ranged from 0.724 to 0.851, indicating a change of −6.58% to a 9.81% increase compared to GBLUP. In the case of CL, the genomic prediction accuracy based on the top 5% to 20% SNPs ranged from 0.667 to 0.673, resulting in an improvement of 18.68% to 19.75% over the traditional GBLUP method. For CD, the genomic prediction accuracy ranged from 0.774 to 0.780, which was 9.32% to 10.17% higher than that achieved using the traditional GBLUP approach.

## 4. Discussion

With the rapid advancement of genomic technologies, GWASs have become a key tool for elucidating the genetic architecture of key economic traits, playing an especially important role in the field of animal breeding [29,30]. Some studies have demonstrated that GWASs not only identify genetic markers associated with economically important traits but also provide valuable biological prior information for GS, thereby improving the accuracy of genomic prediction. In this study, GWAS results were used as prior information to assign weights to variant loci, which were then incorporated into the genomic relationship matrix to enhance the accuracy of genomic estimated breeding values.

The application of genomic prediction technology in animal breeding is becoming more and more extensive, especially in improving the breeding efficiency of important economic traits. Falconer et al. considered heritability values equal to or greater than 0.3 to indicate high heritability, values between 0.1 and 0.3 to indicate moderate heritability, and values below 0.1 to indicate low heritability [31]. In this study, heritability estimates derived from the weighted Gt matrix indicated that WL and CD reflect a high level of genetic influence. CY suggests a moderate to high level of genetic influence. In contrast, the heritability estimates of CL was relatively low. Compared with the results obtained using the GBLUP method, the heritability estimates calculated from the Gt relationship matrix incorporating prior information were higher. In other studies, the heritability estimates for CY in IMCGs ranged from 0.23 to 0.34, while those for CD ranged from 0.27 to 0.36, and for WL from 0.25 to 0.32. All of these fall within the moderate heritability range and are consistent with the findings of the present study. For CL, the heritability estimated in this study was higher than that reported by Rong Youjun [1] (0.05), but lower than those of Fenghong Wang [32], Xuewu Li [33], and Junyan Bai [34] (0.14, 0.17, and 0.21). These differences may be attributed to variations in the number of effective records and the methods used for estimation. Both methods consistently indicated that WL and CD traits have greater potential for genetic improvement.

In this study, the genetic parameters of fleece traits in IMCGs were evaluated by progressively integrating genomic relationship matrices using the top 5–20% of the most significant SNPs from GWAS as prior information. The results showed that as the proportion of top SNPs increased, the amount of genetic variance explained by the prior information matrix also increased. This indicates a cumulative effect of significant SNPs on the genetic variance of the traits [35]. When the top 20% of SNPs were utilized as prior information, the genetic weights for the four fleece traits ranged from 0.72 to 0.80. This further supports the idea that economic traits are regulated by a limited number of genes exerting moderate to large effects. However, when the top set is expanded to capture all the loci associated with the trait, continuing to increase the number of SNPs will hardly increase the genetic variance and heritability, but will lead to a downward trend in the accuracy of GS prediction due to the interference of unrelated loci [36]. Therefore, the prediction accuracy shows a trend of increasing first and then decreasing gradually.

For CY and CL, the highest genomic prediction accuracies were achieved by integrating the top 5% of GWAS-derived SNPs as prior information, with accuracies of 0.742 and 0.673, respectively. This represents improvements of 16.67% and 19.75% over models that did not utilize prior information. For WL and CD, the best prediction accuracies were attained by incorporating the top 10% of prior SNPs, yielding accuracies of 0.851 and 0.780. This corresponds to improvements of 9.81% and 10.14%, respectively, compared to models lacking integrated SNP marker information. Overall, as the number of prior markers increases, the accuracy of GS prediction rises until it reaches a peak, after which it begins to decline. Notably, for the low-heritability trait of CL, prediction accuracy improved significantly. This may be attributed to the prior marker information more comprehensively covering genomic regions harboring minor-effect genes that regulate this trait. In a study on yellow-feathered broiler chickens, Gao et al. [37] constructed haplotypes based on GWAS analysis and incorporated gene annotation information into the genomic selection model, which successfully increased the prediction accuracy for residual feed intake from 0.464 to 0.468, representing an improvement of 0.86%. Li et al. [16], in their study on Alpine Merino sheep, reported that constructing the genomic relationship matrix using 5% to 20% of GWAS-significant loci improved the prediction accuracy of the GBLUP model for 14-month live weight traits by 2.59% to 7.79%. Furthermore, a separate study on sheep demonstrated that the integration of GWAS-derived prior information based on a 50K SNP chip significantly enhanced the genomic prediction accuracy for six meat quality traits and two wool traits [38]. Regarding carcass traits in Hanwoo cattle, Sara de las Heras-Saldana et al. [3] found that the prediction accuracy increased by 2.0% to 5.0% when using GWAS-preselected SNPs compared to the standard 50K SNP panel. Despite potential confounding factors across different studies that may influence the results, it is undeniable that integrating GWAS-derived prior information into genomic prediction models is an effective strategy to enhance the accuracy of genomic prediction.

The results of this study showed that the accuracy of genomic prediction of fleece traits in Inner Mongolia cashmere goats was significantly improved by screening and weighting SNPs as prior information, especially in low heritability traits. In this study, the G-matrix was weighted by integrating the prior site information of GWAS, which provided a new research idea for the genomic breeding of IMCGs. However, due to the significant differences in breeding objectives and genetic background among different breeding populations, population specificity needs to be fully considered in practical applications. Therefore, future research will need to expand the population size and incorporate data from multiple breeds to enhance the universality and applicability of the research findings.

## 5. Conclusions

In this study, SNPs identified through GWAS were ranked based on their *p*-values, and the top 5% to 20% were used as prior information. These SNPs were assigned weights according to their contribution to genetic variance and integrated into a newly constructed genomic relationship matrix for GS. This approach was applied to estimate genetic parameters and evaluate genomic prediction accuracy for fleece traits in IMCGs. The results demonstrate that incorporating pre-selected, functionally relevant SNPs into GS models can significantly enhance the genomic prediction accuracy of fleece traits in IMCGs. This improvement offers valuable support for more precise estimation of genomic breeding values in cashmere goat breeding programs.

## Figures and Tables

**Table 1:** Estimation of genetic parameters for cashmere yield.

Prior Marker	Matrix	$\sigma_{a}^{2}\pm SE$ ^1^	$\sigma_{pe}^{2}\pm SE$ ^2^	$\sigma_{e}^{2}\pm SE$ ^3^	$h^{2}$ ^4^	Rep ^5^	Weight
All	G	7757.99 ± 915.93	5350.80 ± 642.89	25,898.53 ± 442.40	0.20	0.33	
Top 5%	G_1_	8949.80 ± 856.24	3516.57 ± 454.89	25,738.69 ± 437.71	0.23	0.33	0.60
	G_2_	5945.97 ± 823.51	6946.92 ± 674.26	25,876.87 ± 442.27	0.15	0.33	0.40
	G_t_	9757.60 ± 896.64	2682.60 ± 489.95	25,800.89 ± 439.08	0.26	0.33	
Top 10%	G_1_	9474.69 ± 872.38	2826.32 ± 466.42	25,794.13 ± 438.79	0.25	0.32	0.67
	G_2_	4732.10 ± 736.41	8054.56 ± 684.11	25,850.14 ± 441.88	0.12	0.33	0.33
	G_t_	9930.74 ± 905.38	2529.52 ± 497.58	25,833.46 ± 439.78	0.26	0.33	
Top 15%	G_1_	9659.92 ± 887.90	2714.60 ± 482.81	25,831.50 ± 439.62	0.25	0.32	0.71
	G_2_	3921.57 ± 665.65	8825.22 ± 686.01	25,830.28 ± 441.58	0.10	0.33	0.29
	G_t_	9941.97 ± 914.62	2624.22 ± 510.64	25,857.53 ± 440.37	0.26	0.33	
Top 20%	G_1_	9934.10 ± 910.86	2574.76 ± 494.34	25,841.89 ± 439.91	0.26	0.33	0.75
	G_2_	3254.63 ± 598.85	9483.99 ± 684.74	25,812.06 ± 441.27	0.08	0.33	0.25
	G_t_	10,040.40 ± 928.10	2636.01 ± 519.12	25,864.12 ± 440.57	0.26	0.33	

^1^ $\sigma_{a}^{2}$ indicates the genetic variance, ^2^ $\sigma_{pe}^{2}$ indicates the permanent environmental variance, ^3^ $\sigma_{e}^{2}$ indicates the Residual Variance, ^4^ $h^{2}$ indicates heritability, and ^5^ Rep indicates repeatability.

**Table 2:** Estimation of genetic parameters for wool length.

Prior Marker	Matrix	$\sigma_{a}^{2}\pm SE$ ^1^	$\sigma_{pe}^{2}\pm SE$ ^2^	$\sigma_{e}^{2}\pm SE$ ^3^	$h^{2}$ ^4^	Rep ^5^	Weight
All	G	5.93 ± 0.59	3.66 ± 0.34	10.21 ± 0.17	0.30	0.48	
Top 5%	G_1_	7.35 ± 0.58	2.20 ± 0.22	10.16 ± 0.17	0.37	0.48	0.60
	G_2_	4.80 ± 0.54	4.61 ± 0.36	10.22 ± 0.17	0.24	0.48	0.40
	G_t_	7.31 ± 0.57	1.92 ± 0.25	10.17 ± 0.17	0.38	0.48	
Top 10%	G_1_	7.55 ± 0.59	2.05 ± 0.24	10.16 ± 0.17	0.38	0.49	0.65
	G_2_	4.02 ± 0.50	5.25 ± 0.37	10.22 ± 0.17	0.21	0.48	0.35
	G_t_	7.41 ± 0.59	2.01 ± 0.26	10.17 ± 0.17	0.38	0.48	
Top 15%	G_1_	7.33 ± 0.58	2.05 ± 0.25	10.17 ± 0.17	0.37	0.48	0.69
	G_2_	3.36 ± 0.45	5.81 ± 0.38	10.22 ± 0.17	0.17	0.47	0.31
	G_t_	7.28 ± 0.58	2.10 ± 0.27	10.18 ± 0.17	0.37	0.48	
Top 20%	G_1_	7.33 ± 0.58	2.09 ± 0.26	10.17 ± 0.17	0.37	0.48	0.72
	G_2_	2.87 ± 0.41	6.24 ± 0.38	10.22 ± 0.17	0.15	0.47	0.28
	G_t_	7.23 ± 0.59	2.20 ± 0.28	10.18 ± 0.17	0.37	0.48	

^1^ $\sigma_{a}^{2}$ indicates the genetic variance, ^2^ $\sigma_{pe}^{2}$ indicates the permanent environmental variance, ^3^ $\sigma_{e}^{2}$ indicates the Residual Variance, ^4^ $h^{2}$ indicates heritability, and ^5^ Rep indicates repeatability.

**Table 3:** Estimation of genetic parameters for cashmere length.

Prior Marker	Matrix	$\sigma_{a}^{2}\pm SE$ ^1^	$\sigma_{pe}^{2}\pm SE$ ^2^	$\sigma_{e}^{2}\pm SE$ ^3^	$h^{2}$ ^4^	Rep ^5^	Weight
All	G	0.09 ± 0.01	0.00 ± 0.00	0.96 ± 0.01	0.08	0.08	
Top 5%	G_1_	0.10 ± 0.01	0.00 ± 0.00	0.93 ± 0.01	0.09	0.09	0.61
	G_2_	0.06 ± 0.01	0.02 ± 0.01	0.96 ± 0.02	0.06	0.08	0.39
	G_t_	0.10 ± 0.01	0.00 ± 0.00	0.93 ± 0.01	0.09	0.09	
Top 10%	G_1_	0.10 ± 0.01	0.00 ± 0.00	0.93 ± 0.01	0.09	0.09	0.69
	G_2_	0.04 ± 0.01	0.03 ± 0.01	0.96 ± 0.02	0.04	0.07	0.31
	G_t_	0.10 ± 0.01	0.00 ± 0.00	0.93 ± 0.01	0.09	0.09	
Top 15%	G_1_	0.10 ± 0.01	0.00 ± 0.00	0.93 ± 0.01	0.09	0.09	0.75
	G_2_	0.03 ± 0.01	0.04 ± 0.01	0.96 ± 0.02	0.03	0.07	0.25
	G_t_	0.10 ± 0.01	0.00 ± 0.00	0.93 ± 0.01	0.09	0.09	
Top 20%	G_1_	0.10 ± 0.01	0.00 ± 0.00	0.93 ± 0.01	0.09	0.09	0.80
	G_2_	0.02 ± 0.01	0.05 ± 0.01	0.96 ± 0.02	0.02	0.07	0.20
	G_t_	0.10 ± 0.01	0.00 ± 0.00	0.93 ± 0.01	0.09	0.09	

^1^ $\sigma_{a}^{2}$ indicates the genetic variance, ^2^ $\sigma_{pe}^{2}$ indicates the permanent environmental variance, ^3^ $\sigma_{e}^{2}$ indicates the Residual Variance, ^4^ $h^{2}$ indicates heritability, and ^5^ Rep indicates repeatability.

**Table 4:** Estimation of genetic parameters for cashmere diameter.

Prior Marker	Matrix	$\sigma_{a}^{2}\pm SE$ ^1^	$\sigma_{pe}^{2}\pm SE$ ^2^	$\sigma_{e}^{2}\pm SE$ ^3^	$h^{2}$ ^4^	Rep ^5^	Weight
All	G	0.14 ± 0.01	0.04 ± 0.01	0.33 ± 0.01	0.28	0.36	
Top 5%	G_1_	0.18 ± 0.02	0.01 ± 0.01	0.32 ± 0.01	0.35	0.37	0.61
	G_2_	0.12 ± 0.01	0.06 ± 0.01	0.32 ± 0.01	0.23	0.35	0.39
	G_t_	0.18 ± 0.02	0.00 ± 0.01	0.32 ± 0.01	0.35	0.36	
Top 10%	G_1_	0.18 ± 0.02	0.01 ± 0.01	0.33 ± 0.01	0.35	0.36	0.65
	G_2_	0.09 ± 0.01	0.08 ± 0.01	0.32 ± 0.01	0.19	0.35	0.35
	G_t_	0.17 ± 0.01	0.00 ± 0.01	0.32 ± 0.01	0.35	0.35	
Top 15%	G_1_	0.17 ± 0.01	0.01 ± 0.01	0.33 ± 0.01	0.34	0.35	0.70
	G_2_	0.07 ± 0.01	0.10 ± 0.01	0.32 ± 0.01	0.15	0.34	0.30
	G_t_	0.17 ± 0.01	0.00 ± 0.01	0.32 ± 0.01	0.35	0.35	
Top 20%	G_1_	0.17 ± 0.01	0.01 ± 0.01	0.33 ± 0.01	0.34	0.35	0.74
	G_2_	0.06 ± 0.01	0.11 ± 0.01	0.32 ± 0.01	0.12	0.34	0.26
	G_t_	0.17 ± 0.01	0.00 ± 0.01	0.32 ± 0.01	0.34	0.35	

Genomic Prediction Accuracy Using a GWAS-Based Weighted Matrix. ^1^ $\sigma_{a}^{2}$ indicates the genetic variance, ^2^ $\sigma_{pe}^{2}$ indicates the permanent environmental variance, ^3^ $\sigma_{e}^{2}$ indicates the Residual Variance, ^4^ $h^{2}$ indicates heritability, and ^5^ Rep indicates repeatability.

**Table 5:** Genomic prediction accuracy for fleece traits.

Prior Marker	CY		WL		CL		CD	
	Accuracy	Promotion ^2^	Accuracy	Promotion	Accuracy	Promotion	Accuracy	Promotion
All	0.636 ^b **1**^		0.775 ^b^		0.562 ^b^		0.708 ^b^	
Top 5%	0.742 ^a^	16.67%	0.785 ^b^	1.29%	0.673 ^a^	19.75%	0.776 ^a^	9.60%
Top 10%	0.741 ^a^	16.51%	0.851 ^a^	9.81%	0.673 ^a^	19.75%	0.780 ^a^	10.17%
Top 15%	0.735 ^a^	15.57%	0.745 ^c^	−3.87%	0.669 ^a^	19.04%	0.776 ^a^	9.60%
Top 20%	0.732 ^a^	15.09%	0.724 ^cd^	−6.58%	0.667 ^a^	18.68%	0.774 ^a^	9.32%

^1^ Different letters indicate significant difference at *p* < 0.05, and the same letters indicate insignificant difference at *p* > 0.05. ^2^ Promotion is used to quantify the degree of improvement in predictive performance of the G_t_ matrix relative to the individual G_1_ and G_2_ matrices.

## Data Availability

The phenotypic data of Inner Mongolia cashmere goats used in this study contain commercial confidentiality of the breeding farms and are not publicly available. However, the de-identified phenotypic data (with farm identifiers removed) and genotypic data (after quality control, 67,021 SNPs) are available on request from the corresponding author due to restrictions on commercial data sharing. The genotypic data have been deposited in the Figshare repository with the DOI: https://doi.org/10.6084/m9.figshare.30413845.

## Abbreviations

The following abbreviations are used in this manuscript:

IMCG	Inner Mongolia Cashmere goats
CY	Cashmere yield
WL	Wool length
CL	Cashmere length
CD	Cashmere diameter
GWAS	Genome-wide association study
GS	Genomic selection
GEBV	Genomic estimated breeding value
GBLUP	Genomic best linear-unbiased prediction
G-matrix	Genomic relationship matrix
GFBLUP	Genomic Feature Best Linear Unbiased Prediction
MA-BLUP	Marker-assisted BLUP
BLUE	Best Linear Unbiased Estimates
BLINK	Linkage-disequilibrium Iteratively Nested Keyway
