# Correction: Yao et al. Genome Selection for Fleece Traits in Inner Mongolia Cashmere Goats Based on GWAS Prior Marker Information. *Animals* 2025, *15*, 3184

**DOI:** 10.3390/ani16010102

**Published:** 2025-12-30

**Authors:** Huanfeng Yao, Na Wang, Yu Li, Gang He, Jin Ning, Shuai Kang, Yongbin Liu, Jinquan Li, Qi Lv, Ruijun Wang, Yanjun Zhang, Rui Su, Zhiying Wang

**Affiliations:** 1 College of Animal Science, Inner Mongolia Agricultural University, Hohhot 010018, China; 2 Inner Mongolia Yiwei White Cashmere Goat Co., Ltd., Ordos 017000, China; 3 Inner Mongolia Key Laboratory of Sheep & Goat Genetics Breeding and Reproduction, Hohhot 010018, China; 4 Key Laboratory of Mutton Sheep & Goat Genetics and Breeding, Ministry of Agriculture and Rural Affairs, Hohhot 010018, China

## Text Correction

There was an error in the original publication [1]. Due to the authors’ oversight, a data presentation mistake has occurred. The pre-experiment of this study set the analysis interval as “Top 5%–Top 30%”, while the actual presented results correspond to an interval of “Top 5%–Top 20%”. However, the result description incorrectly adopted the original interval, which has now been uniformly corrected to “Top 5%–Top 20%”. The original publication has also been updated. A correction has been made to *3. Results*, *3.2. Estimates of Genetic Parameters of Fleece Traits in IMCGs*, Paragraph 5. The corrected paragraph is provided below.

By incorporating the top 5% to 20% of GWAS-identified SNPs as prior marker information into the genomic relationship matrix (Table 5 and Figure 3), the genomic prediction accuracy for CY ranged from 0.732 to 0.742, representing an improvement of 15.09% to 16.67% over the traditional GBLUP method. For WL, the genomic prediction accuracy with GWAS-informed prior markers ranged from 0.724 to 0.851, indicating a change of −6.58% to a 9.81% increase compared to GBLUP. In the case of CL, the genomic prediction accuracy based on the top 5% to 20% SNPs ranged from 0.667 to 0.673, resulting in an improvement of 18.68% to 19.75% over the traditional GBLUP method. For CD, the genomic prediction accuracy ranged from 0.774 to 0.780, which was 9.32% to 10.17% higher than that achieved using the traditional GBLUP approach.

## Error in Figure

In the original publication [1], there was a mistake in Figure 3 as published. The image in Figure 3 contained an incorrect data panel that corresponded to the “Top 5%–Top 30%” interval instead of the intended “Top 5%–Top 20%” interval. The corrected Figure 3 is provided below.

**Figure 3:**
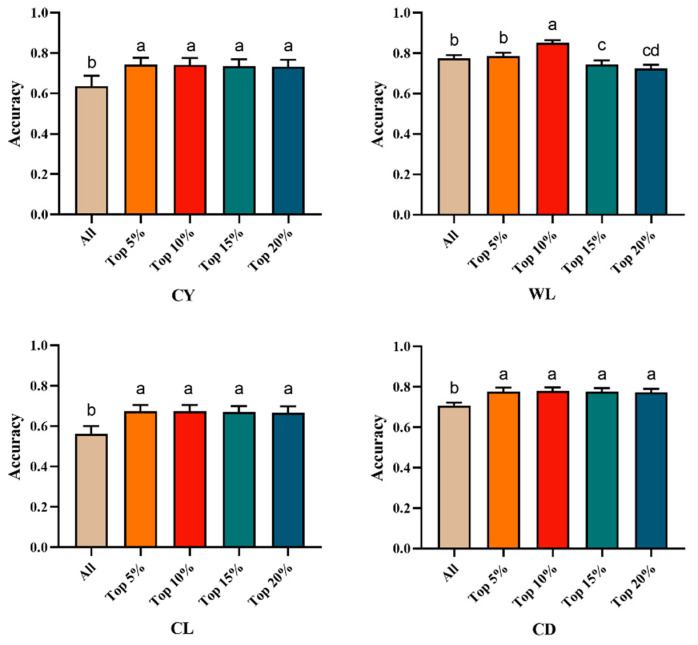
Multiple comparison plots of genome prediction accuracy for fleece traits with integrated Top prior information. Different letters indicate a significant difference at *p* < 0.05, and the same letters indicate no significant difference at *p* > 0.05. CY = cashmere yield; WL = wool length; CL = cashmere length; and CD = cashmere diameter.

The authors state that the scientific conclusions are unaffected. This correction was approved by the Academic Editor. The original publication has also been updated.
